# A novel epithelial–mesenchymal transition gene signature for the immune status and prognosis of hepatocellular carcinoma

**DOI:** 10.1007/s12072-022-10354-3

**Published:** 2022-06-14

**Authors:** Yanlong Shi, Jingyan Wang, Guo Huang, Jun Zhu, Haokun Jian, Guozhi Xia, Qian Wei, Yuanhai Li, Hongzhu Yu

**Affiliations:** 1 Department of General Surgery, Fuyang Hospital Affiliated to Anhui Medical University, Fuyang, 236000 Anhui China; 2 Department of Anesthesiology, Chaohu Hospital Affiliated to Anhui Medical University, Chaohu, 238000 Anhui China; 3 Hengyang Medical College, University of South China, Hengyang, 421001 Hunan China; 4 Key Laboratory of Tumor Cellular and Molecular Pathology, Cancer Research Institute, College of Hunan Province, University of South China, Hengyang, 421001 Hunan China; 5 Department of Oncology, Fuyang Hospital Affiliated to Anhui Medical University, Fuyang, 236000 Anhui China; 6 School of Basic Medical Sciences, Xinxiang Medical University, Xinxiang, 453000 Henan China; 7 School of Nursing, Anhui Medical University, HeFei, 230000 Anhui China

**Keywords:** Model, Decision-making, Epithelial–mesenchymal transition, Hepatocellular carcinoma, Prognosis, Immune microenvironment, Bioinformatics, Overall survival, Drug sensitivity, Biomarker

## Abstract

**Background:**

This study clarified whether EMT-related genes can predict immunotherapy efficacy and overall survival in patients with HCC.

**Methods:**

The RNA-sequencing profiles and patient information of 370 samples were derived from the Cancer Genome Atlas (TCGA) dataset, and EMT-related genes were obtained from the Molecular Signatures database. The signature model was constructed using the least absolute shrinkage and selection operator Cox regression analysis in TCGA cohort. Validation data were obtained from the International Cancer Genome Consortium (ICGC) dataset of patients with HCC. Kaplan–Meier analysis and multivariate Cox analyses were employed to estimate the prognostic value. Immune status and tumor microenvironment were estimated using a single-sample gene set enrichment analysis (ssGSEA). The expression of prognostic genes was verified using qRT-PCR analysis of HCC cell lines.

**Results:**

A signature model was constructed using EMT-related genes to determine HCC prognosis, based on which patients were divided into high-risk and low-risk groups. The risk score, as an independent factor, was related to tumor stage, grade, and immune cells infiltration. The results indicated that the most prognostic genes were highly expressed in the HCC cell lines, but GADD45B was down-regulated. Enrichment analysis suggested that immunoglobulin receptor binding and material metabolism were essential in the prognostic signature.

**Conclusion:**

Our novel prognostic signature model has a vital impact on immune status and prognosis, significantly helping the decision-making related to the diagnosis and treatment of patients with HCC.

**Supplementary Information:**

The online version contains supplementary material available at 10.1007/s12072-022-10354-3.

## Background

Liver cancer is the fourth leading cause of cancer-related deaths and the sixth most common malignancy worldwide, with more than 900,000 newly cases and an estimated 800,000 deaths in 2020 [1]. Hepatocellular carcinoma (HCC), the most common type of primary liver cancer, is mainly caused by viral hepatitis, alcohol abuse, moldy food consumption, and genetic factors [2]. The prognosis of HCC mainly contributes to early diagnosis, which ensures that effective treatment measures are taken. However, a growing number of patients have advanced stages when they are first diagnosed, bringing great challenges to the treatment of HCC [3]. High-level heterogeneity, along with the aggressive ability of HCC, induces poor prognosis, with a 5-year survival rate of only 14% [4]. Therefore, it is imperative to develop novel strategies to prolong survival time and provide guidance for the individualized treatment of patients with HCC.

EMT is a multi-step cell biology process that drives the reversible dedifferentiation of epithelial cells into a mesenchymal-like or mesenchymal phenotype to induce embryogenesis, wound healing, and tumorigenesis [5, 6]. The tumor microenvironment (TME) related to tumor cell reprogramming promotes tumor progression and drug resistance through the malignant transformation of EMT [7, 8]. Previous studies have shown that EMT strongly participated in the occurrence and progression strongly in patients of HCC [9, 10]. Currently, increasing attention has been given to a signature designed for forecasting the prognosis of cancer and exploring its underlying mechanism using public database information. For example, Zhao et al. constructed a cycle-related gene signature to identify the prognosis of gastric cancer and further explore its cell cycle mechanisms [11]. Moreover, Lin et al. showed that a prognostic signature related to inflammatory responses influences immune status and survival prognosis [12]. However, there have been limited reports on novel signature models of EMT-related genes that can predict the prognosis of patients with HCC.

In this study, we obtained the RNA-sequencing profiles and patient information of 370 HCC samples from TCGA dataset, as well as obtaining EMT-related genes from the Molecular Signatures database. Subsequently, based on EMT-related differentially expressed genes (DEGs), we manufactured a novel prognostic signature using the TCGA as the training cohort and ICGC as the validation cohort. We divided DEGs into high-risk and low-risk groups by risk score to confirm the relationship between the prognostic model, clinicopathological features, and immune microenvironment. Moreover, an enrichment analysis was performed to investigate the molecular mechanisms. Additionally, we demonstrated a connection between prognostic genes and drug sensitivity in the NCI-60 database. Finally, we verified the expression of the mRNA of these prognostic genes in the HCC cell lines.

## Methods

### Data collection

The RNA-sequencing profiles and patient information of 370 HCC samples were downloaded from the TCGA database; these samples were included to the training cohort (https://portal.gdccancer.gov/repository). The validation cohort data for this study were extracted from 231 patients with HCC and their corresponding clinical information in the ICGC database (https://dcc.icgc.org/). Moreover, the Molecular Signatures database was used to investigate EMT-related genes (http://www.gsea-msigdb.org/gsea/; systematic name: M5930). The results are demonstrated in Supplementary Table 1. The obtained public data comply with the database access policies and publication guidelines of the aforementioned databases.

### Construction and validation of an EMT-related gene signature

The DEGs of HCC were identified by R package "limma" in tumor and corresponding non-tumor tissues of the TCGA cohort with |log_2_FC|> 2, and FDR < 0.05. A univariate Cox regression analysis was performed for DEGs. Patients from the TCGA cohort were assigned to the training group, whereas those from the ICGC database were assigned to the validation group in a 1:1 manner. The Lasso Cox regression algorithm and the "glmnet" R package were used to minimize the risk of overfitting, and the EMT-related gene model was established using tenfold cross-validation [13, 14]. The independent variable was candidate genes for the prognostic model in the regression analysis, and the dependent variable was overall survival (OS) or survival status in patients with HCC in the TCGA cohort. A multivariate Cox analysis was used to select candidate genes and establish an EMT-related risk score [15]; the formula was as follows: risk score = e^(…. corresponding coefficient + …. + SPP1 expression), with Coefi and Expi representing the risk coefficient and expression level of each gene, respectively. Based on the median risk score, patients were divided into the high-risk and low-risk groups. The R package "survminer" was used to estimate OS by a survival analysis in both the high-risk and low-risk groups. The R package "timeROC" was used to evaluate the prognostic value of the signature model. Moreover, univariate Cox analyses, multivariate Cox analyses, and the R package "survival" were used to determine whether the signature was an independent prognostic factor. Principal component analysis (PCA) and t-distributed random neighbor embedding (t-SNE) analyses were employed to investigate the distribution of groups using the “Rtsne” and “ggplot2” R packages.

### Functional enrichment analysis

To further explore the results of the functional analysis, we determined to distinguish DEGs in the low-risk and high-risk groups using the R packages “limma”. The thresholds were as follows: Mann–Whitney test, |log2FC|≥ 1, and *p* value < 0.05. The R package “clusterProfiler” was utilized to analyze gene ontology (GO) and Kyoto Encyclopedia of Genes and Genomes (KEGG).

### Tumor microenvironment analysis

The infiltration of immune and stromal cells can be obtained by analyzing the immune and stromal scores of different tumors. The relationship between the risk, immune, and stromal scores was detected using Spearman’s correlations. A two-way analysis of variance (ANOVA) was used to explore the correlation between the risk score and immune subtypes. Spearman’s correlation was also applied to observe the correlation between stem cell-like features of TCGA transcriptome tumors and risk scores.

### Drug sensitivity analysis

There are 60 different cancer cell lines from 9 different tumors comprising the NCI-60 CellMiner database (https://discover.nci.nih.gov/cellminer). The relationship between the prognostic signature of EMT-related genes and drug sensitivity was conducted using Pearson’s correlation analysis.

### Cell culture

Human HCC cell lines (Huh-7 and HepG2) and human hepatic epithelial cell (LO2) were presented from central laboratory of the First Hospital Affiliated to Anhui Medical University who purchased from Procell Life Science (Wuhan, China), and cultured in high-glucose DMEM containing 10% fetal bovine serum (HyClone; VivaCell, Shanghai, China). The cells were subsequently incubated at 37 °C in a 5% CO_2_ incubator.

### Quantitative real-time polymerase chain reaction (qRT-PCR)

Total RNA was extracted from cells using the TRIzol reagent (Takara). To quantify prognostic gene levels, a reverse transcription of cDNA was performed using the PrimeScript™ kit (Takara). Prognostic gene expression levels were measured using the SYBR Green qPCR Mix (Takara). The relative expression of prognostic genes was determined using the 2^−ΔΔCt^ method. Primer sequences are listed in Supplementary Table 2.

### Statistical analysis

The Wilcoxon test was applied to distinguish DEGs between HCC tumors and normal tissues. Different proportions were detected using the Chi-squared test. The Mann–Whitney test was used to assess ssGSEA scores of the immune microenvironment between the high-risk and low-risk groups. The R packages “venn”, “pheatmap”, “graph”, “ggplot2”, “ggpubr”, “corrplot”, and “survminer” were used to plot maps using the R software version 3.8. A one-way ANOVA was used to compare the expression between the human HCC cell line and human hepatic epithelial cells. A two-tailed *p* value < 0.05 was considered statistically significant.

## Results

We investigated the TCGA-LIHC cohort (365 patients with HCC) and the ICGC cohort (231 HCC patients with HCC).

### Identification and construction of EMT-related prognostic signature in the TCGA cohort

**Fig. 1:**
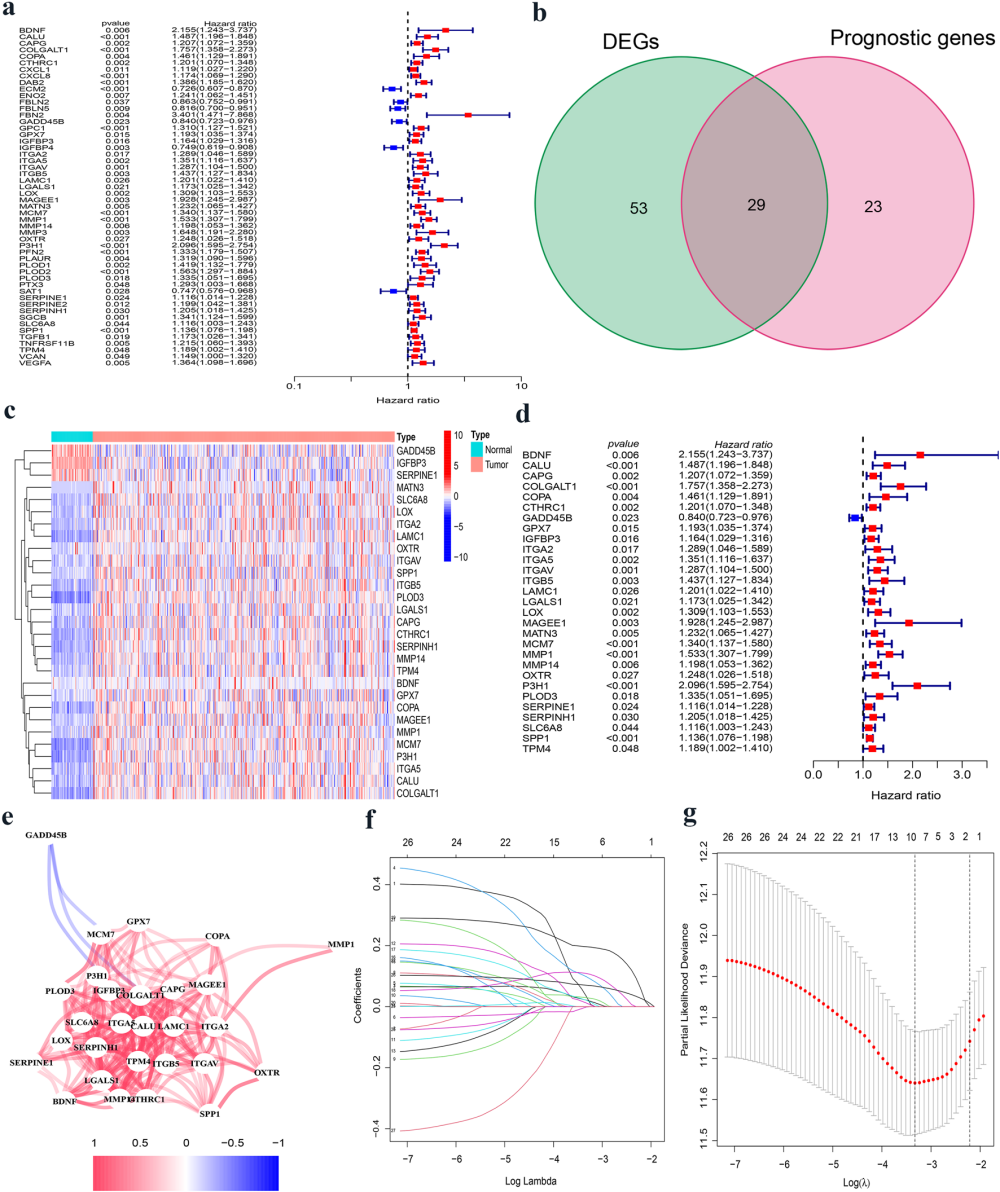
Establishment of the EMT-related prognostic signature in the TCGA cohort. **a** The forest plots showing the association between 52 prognostic genes expression and OS. **b** Venn diagram to distinguish DEGs between HCC and adjacent normal tissues. **c** Heatmap of the 29 overlapping genes expression. **d** Univariate Cox regression analysis of 29 overlapping genes associated with OS. **e** The correlation network of prognostic genes signature. **f** LASSO coefficient profiles of 29 prognostic genes of HCC. **g** LASSO regression with tenfold cross-validation found ten prognostic genes using the minimum λ

There are 52 differentially expressed genes between HCC tissues and normal tissues (Fig. 1a). The differential expression of 81 EMT-related genes was identified in tumor and normal tissues. Using a univariate Cox analysis, we found that 29 of the 52 genes were interconnected with OS (Fig. 1b). These 29 candidate genes were considered as prognostic factors (Fig. 1c, d). The relationships between candidate genes are shown in Fig. 1e. A prognostic signature was built for these genes using the LASSO regression.

**Fig. 2:**
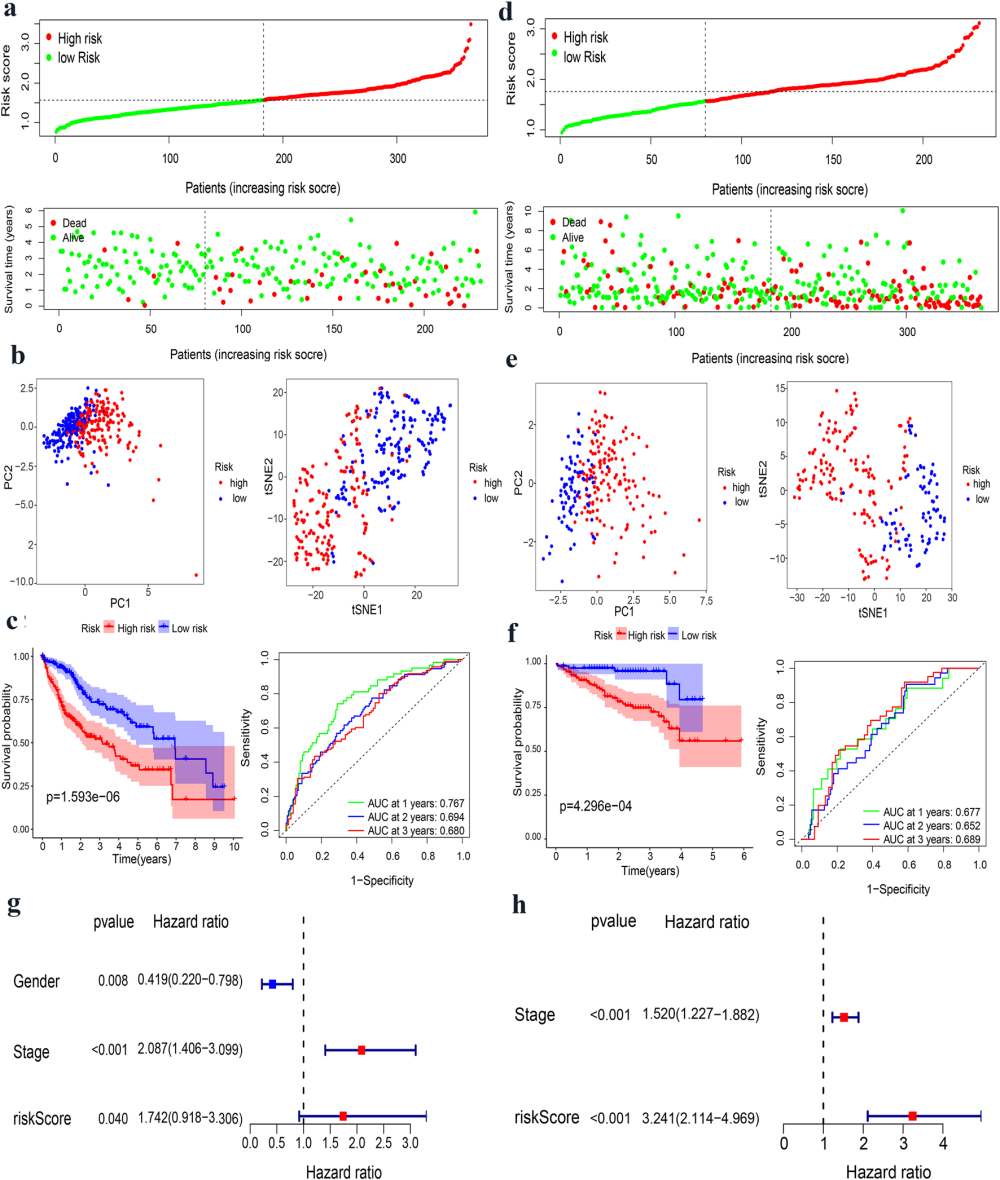
Evaluation and validation of 10-gene signature in TCGA cohort and ICGC cohort. **a** Analysis of risk score value and distribution, OS status, and heatmap of 10-gene signature model in TCGA cohort. **b** The PCA plot and t-SNE analysis of risk score in TCGA cohort. **c** Kaplan–Meier curves and AUC time-dependent ROC curves for OS in TCGA cohort. **d** Analysis of risk score value and distribution, OS status, and heatmap of 10-gene signature model in ICGC cohort. **e** The PCA plot and t-SNE analysis of risk score in ICGC cohort. **f** Kaplan–Meier curves and AUC time-dependent ROC curves for OS in ICGC cohort. **g, h** Screening of OS-related pathological feature by multivariate Cox regression in TCGA and ICGC cohort

A LASSO regression analysis was used to build prognostic models for the candidate genes to address collinearity. We identified a signature of 10 EMT-related genes based on the optimal cut-off threshold of λ (Fig. 1f, g). The risk score was as follows: risk score = 0.0344*(BDNF expression) + 0.0985*(COPA expression) + (− 0.0011)*(GADD45B expression) + 0.0215*(GPX7 expression) + 0.0122*(ITGB5 expression) + 0.0295*(LOX expression) + 0.0211*(MATN3 expression) + 0.1091*(MCM7 expression) + 0.1965*(MMP1 expression) + 0.0705*(SPP1 expression). Based on the median cut-off threshold, the TCGA cohort comprising patients with HCC was divided into the high-risk and low-risk groups (Fig. 2a). Furthermore, we observed that risk score of patients in the high-risk group was related to tumor grade and TNM stage (Table 1). Using PCA and t-SNE algorithms, we demonstrated that patients were dispersed in various directions in both the low- and high-risk groups (Fig. 2b). According to the scatter plot, compared with the low-risk group, the survival time of patients with HCC in the high-risk group was significantly reduced, which was consistent with the result of the Kaplan–Meier curve (*p* < 0.001). Besides, ROC curves were conducted to forecast the overall survival of the prognostic model, and the area under the curve (AUC) for the 1-, 2-, and 3-year OS were 0.767, 0.694, and 0.680, respectively, in the TCGA cohort (Fig. 2c). Moreover, we investigated the expression of each prognostic gene, which suggested that most genes were highly expressed in HCC tissues compared to normal tissues, yet the opposite was true for GADD45B (Supplementary Fig. 1A–J). In addition, the results of the survival analysis disclosed that the low expressions of BNDF, GPX7, LOX, MATN3, MCM7, MMP1, and SPP1 were interrelated with longer OS (Supplementary Fig. 2a–j).

**Table 1:** Baseline characteristics of the HCC patients in different risk groups

Characteristics	TCGA-LIHC cohort			ICGC-LIHC cohort		
	High risk	Low risk	*p* value	High risk	Low risk	*p* value
Age						
≤65 year	110 (60.44%)	117 (63.93%)	0.5616	54 (35.76%)	35 (43.75%)	0.296
>65 year	72 (39.56%)	66 (36.07%)		97 (64.24%)	45 (56.25%)	
Gender						
Female	54 (29.67%)	65 (35.52%)	0.28	39 (25.83%)	22 (27.5%)	0.9065
Male	128 (70.33%)	118 (64.48%)		112 (74.17%)	58 (72.5%)	
Grade						
G1–2	101 (55.49%)	129 (70.49%)	0.0048			
G3-4	78 (42.86%)	52 (28.42%)				
Unknown	3 (1.65%)	2 (1.09%)				
Stage						
I–II	113 (62.09%)	141 (77.05%)	0.0038	79 (52.32%)	62 (77.5%)	3.00E-04
III–IV	55 (30.22%)	32 (17.49%)		72 (47.68%)	18 (22.5%)	
Unknown	14 (7.69%)	10 (5.46%)		0	0	

### Validation of the EMT-related gene signature in the ICGC cohort

The same calculation was applied to the ICGC cohort to validate the availability of prognostic signature of the TCGA cohort. The ICGC cohort was classified into low-risk and high-risk groups according to the median cut-off value of the risk score (Fig. 2d). Analogously, the risk group distribution, PCA algorithms, t-SNE algorithms, survival status, and Kaplan–Meier curve were also presented. The result of the ICGC queue was the same as that of the TCGA queue (Fig. 2e). Moreover, the AUC of the EMT-related signature was 0.677, 0.652, and 0.68 for the 1 -, 2 -, and 3-year OS, respectively (Fig. 2f). Taken together, our results demonstrate that the EMT-related prognostic signature model could distinguish favorable prognosis in patients with HCC.

### Independent factor of the EMT-related prognostic signature

In the TCGA cohort, the risk score was associated with OS by a multivariate cox analysis, and the risk score was still an independent factor of OS, regardless of the TCGA cohort (HR = 1.742, 95% CI = 0.918–3.306, *p* < 0.05) or in the ICGC cohort (HR = 3.241, 95% CI = 2.114–4.969, *p* < 0.001) (Fig. 2g, h). Hence, combined with the risk score and clinicopathological characteristics, the EMT-related gene signature can better represent the prognosis of patients with HCC.

### Clinicopathological characteristics and prognostic signature risk score

**Fig. 3:**
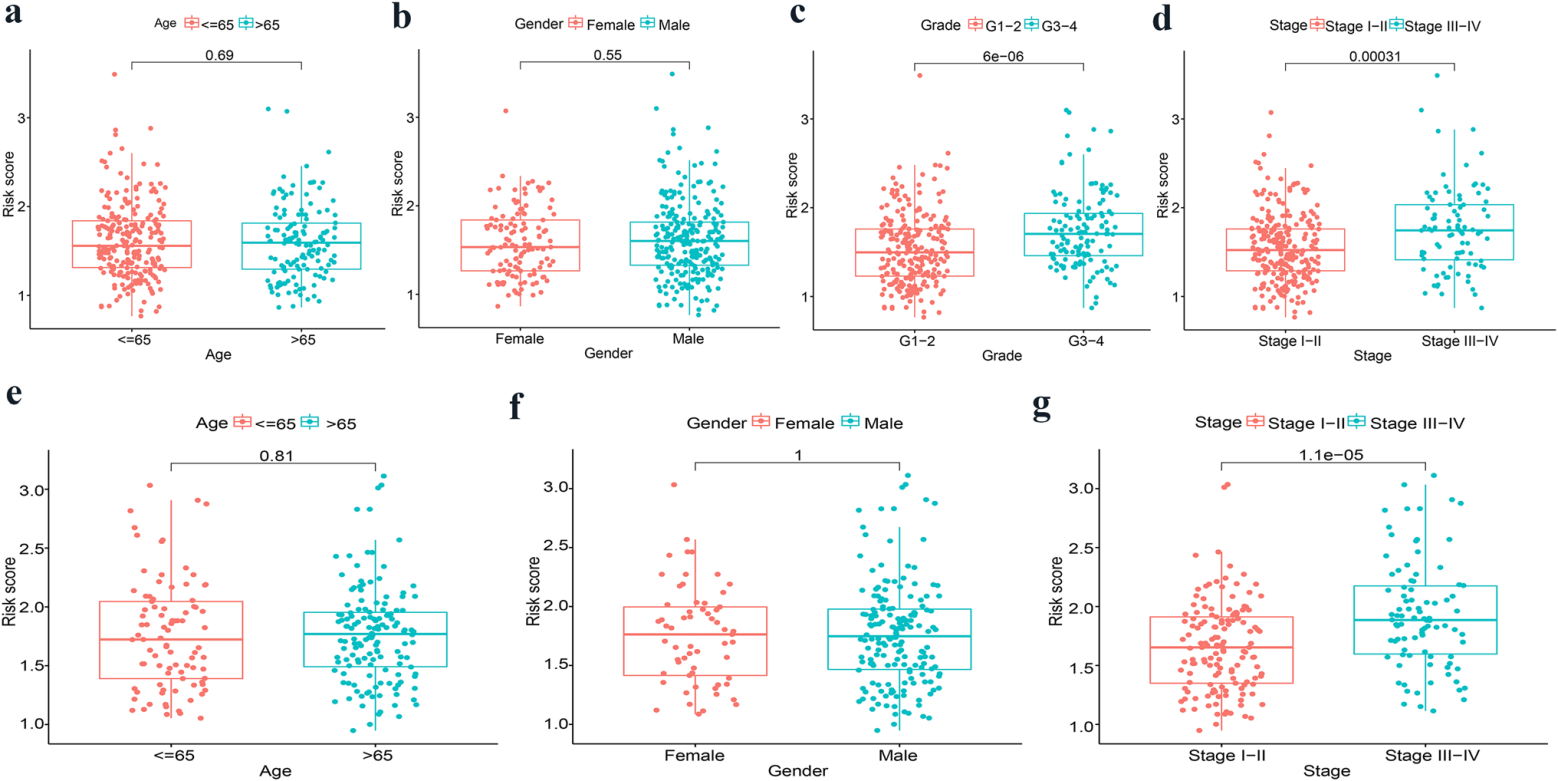
Relationship between risk score and clinicopathologic characteristics. TCGA cohort: **a** Age. **b** Gender. **c** Tumor grade. **d** Tumor stage. ICGC cohort: **e** Age. **f** Gender. **g** Tumor stage

To further certify the value of the EMT-related prognostic signature, the relationship between clinicopathological characteristics and risk score was investigated. We demonstrated that the risk score was concerned with tumor stage and tumor grade in the TCGA cohort (*p* < 0.001), regardless of age and gender (*p* > 0.05) (Fig. 3a–d). And the higher the tumor stage and grade, the higher is score. Similarly, there was a higher risk score for patients having tumor stages III–IV in contrast to patients having lower stages I–II in the ICGC cohort (*p* < 0.001) (Fig. 3e–g). However, HCC tumor grade data were not available for the ICGC cohort.

### Analysis of immune status and immune microenvironment

**Fig. 4:**
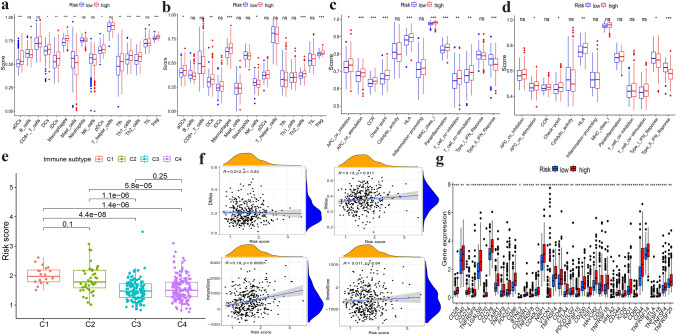
Evaluation immune status, tumor microenvironment, and immune checkpoints of EMT-related prognostic signature. **a, b** The scores of 16 immune cells and 13 immune-related functions were detected by ssGSEA analysis based on risk groups in TCGA cohort and ICGC cohort. **c, d** The scores of 16 immune cells and 13 immune-related functions were detected by ssGSEA analysis based on risk groups in TCGA cohort and ICGC cohort. **e** Risk score of different immune infiltration subtypes. **f** The correlation between risk score and RNAss, DNAss, Stromal Score, and Immune Score. **g** Expression of immune checkpoint genes in high- and low-risk groups. *p < 0.05; **p < 0.01; ***p < 0.001

We performed an ssGSEA analysis to observe immune cell subpopulations, immune functions, and pathways in the high-risk and low-risk groups or risk scores. The results showed that the immune cells including aDS (adipose-derived stem), macrophages, Th1-cells, Th2-cells, and Tregs were markedly expressed in the high-risk group of the TCGA cohort (*p* < 0.001) (Fig. 4a,b). Regarding immune function, the risk levels of APC co-stimulation, CCR (chemokine receptor), checkpoint, HLA, MHC-class-I, and parainflammation were higher in the low-risk group than in the high-risk group, while the functionality of the Type-II-INF-response was opposite to these functions (Fig. 4c, d). There were no statistically significant differences in cytolytic activity or type I IFN responses between groups. The relationship between other markers related to the immune system and the risk score was consistent in the TCGA database, except for iDCs (immature dendritic cells) and Th1-cells.

Furthermore, we explored the association between immune infiltrates and risk score, aiming to reveal the specific role of EMT-related gene signatures in the immune microenvironment. There were six types of immune infiltrates in human tumors, namely C1 (wound healing), C2 (INF-G dominance), C3 (inflammatory), C4 (lymphodepletion), C5 (immune silence), and C6 (TGF-B dominance) [16].

Due to the absence of the C5 and C6 subtypes in patients with HCC, we detected the left subtypes. As shown in Fig. 4e, immune-infiltrating subtypes, especially the C1 and C2 subtypes, were strongly associated with high-risk scores in the TCGA database, indicating that the expression of the EMT-related gene signature has an effect on immune infiltrates in patients with HCC.

Tumor stemness was mainly composed of the RNA stemness score (RNAss) and DNA stemness score (DNAss), reflecting the dedifferentiation characteristics of cancers. Immune and stromal cells are the primary types of non-tumor components, and have been proposed to be valuable for tumor diagnosis and prognosis evaluation. Immune and stromal cell scores were calculated to predict immune cell infiltration by analyzing the specific gene expression features of immune cells and stromal cells. We found that the risk score was positively correlated with RNAss and immune score (*p* < 0.05), but not with DNAss (*p* = 0.83) or stromal scores (*p* = 0.84) (Fig. 4f).

The expression of immune checkpoints plays a crucial role in the prognosis and treatment of cancer. We first explored the association between the risk groups and immune checkpoints (Fig. 4g). Thereafter, we demonstrated that the expression of immune checkpoints, including PD-L1, CTLA4, CXCR2, and TLR8, significantly increased in the high-risk group in the TCGA cohort (Supplementary Fig. 3A–D). In addition, the high-risk group was positively associated with the expression of these immune checkpoints, indicating that the risk score highlights the capacity for immune evasion (Supplementary Fig. 3E–H).

### Analysis of biological function and pathway

**Fig. 5:**
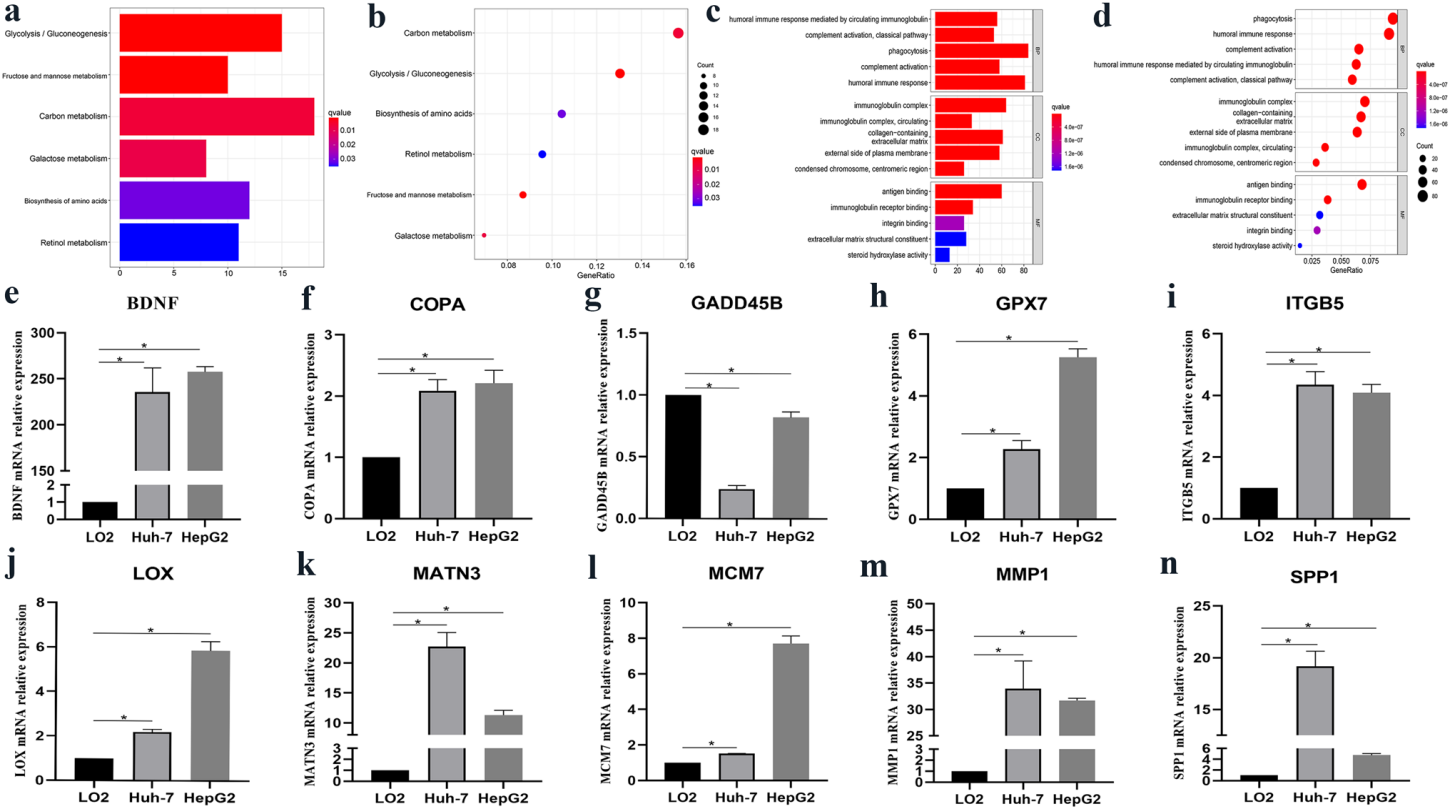
Gene enrichment analysis for high-risk and low-risk groups. **a** KEGG pathway by barplot. **b** KEGG pathway by bubble plot. **c** Gene Ontology by barplot. **d** Gene Ontology by bubble plot. Verification of the expression of EMT-related prognostic genes mRNA in HCC cell line by qRT-RCR. **e** BDNF. **f** COPA. **g** GADD45B. **h** GPX7. **i** ITGB5. **j** LOX. **k** MANT3. **l** MCM7. **m** MMP1. **n** SPP1. *p < 0.05

To explore the underlying functions and mechanisms of the prognostic signature, we conducted GO function and KEGG analyses based on the high-risk and low-risk groups. According to the enrichment score, the results with the top terms are presented in the bar plot and bubble plot. The GO analysis suggested that leukocyte migration, phagocytosis, and human immune response were extensively enriched in biological processes. The primary enrichment of cellular components was the immunoglobulin complex, collagen-containing extracellular matrix, and external side of the plasma membrane. Antigen, cell adhesion molecule binding, and immunoglobulin receptor binding were the first three enriched targets (Fig. 5a, b). In addition, KEGG pathways indicated that carbon metabolism, glycolysis/gluconeogenesis, and biosynthesis of amino acids were the top three enriched terms, suggesting that the prognostic signature was closely related to material metabolism (Fig. 5c, d).

### Relationship between prognostic signature and drug sensitivity

We used the NCI-60 cell line to investigate the relationship between prognostic gene expression and drug sensitivity. These results suggest that GPX7, MATN3, GADD45B, ITGB5, BDNF, MMP1, and LOX are interrelated with the drug sensitivity of chemotherapy. On one hand, the increased expression of GPX7 and LOX was associated with the chemotherapy resistance of cancer cells to Fluphenazine, arsenic trioxide, nellarabine, erlotinib, and lenvatinib (Supplementary Fig. 4A). However, the increased expression of GADD45B, ITGB5, MMP1, and BNDF was correlated with a decreased chemotherapy resistance of cancer cells to mithramycin, tramrtinib, ARRY-162, dabrafenib, selumetinib, vemurafenib, nilotinib, coimetinib, cyclophosphamide, oxaliplatin, and tamoxifen (Supplementary Fig. 4B). Notably, an increased expression of MATN3 was negatively correlated with the drug resistance of cancer cells to eribulin mesylate, vinblastine, pipobroman, paclitaxel, and erlotinib.

### Experimental verification of the prognostic signature expression in HCC cell line

To verify the expression of EMT-related prognostic signatures, including BDNF, COPA, GADD45B, GPX7, ITGB5, LOX, MATN3, MCM7, MMP1, and SPP1, in HCC cell lines, we conducted a qRT-PCR analysis to determine mRNA expression. Prognostic genes, except for GADD45B, were up-regulated in Huh and HepG2 cell lines compared to the LO-2 cell line (*p* < 0.05) (Fig. 5e–n). These results were in accordance with the mRNA expression profiles of these genes in the TCGA cohort.

## Discussion

Liver cancer is a major public health problem in humans. Because of the strong invasion and metastatic capacity of HCC, most patients are at an advanced stage when diagnosed, resulting in a poor prognosis and a 5-year survival rate of < 15% [4]. Recently, with the rapid development of genomics, high-throughput sequencing, and other scientific technologies, the identification of genes with potential prognosis is an urgent problem. Dai et al. built an immune-related gene signature that could predict survival and immunotherapeutic effects [17]. Deng et al. constructed a prognostic signature of HCC subtypes based on a ferroptosis phenotype-related clinical-molecular analysis. This model can stratify patients for clinical decision-making [18]. EMT is the initiating factor for tumor invasion and metastasis. When EMT occurs in tumor cells, the adhesion between tumor cells is weakened, and a loss of cell polarity and cytoskeletal changes occur; this is conducive to cell metastasis [19]. Studies have shown that the poor prognosis of patients with lung, prostate and bladder cancers may be attributed to EMT [20–22]. Previous studies have reported that single prognostic markers associated with EMT in patients with HCC [23, 24]. However, during the occurrence and development of EMT, it acts a pivotal role in tumor invasion and metastasis not only as a differentiation marker, but also in cell morphology and cell function, which involves many gene-level changes. The prediction of the prognosis of patients with HCC based on EMT-related gene signatures has not been illustrated. Therefore, our study established an EMT-related gene signature by simulating the risk score, which can not only predict the prognosis of patients with HCC more accurately, but also provide guidance for individual treatment of patients.

The prognostic model raised in this study comprised ten EMT-related genes, including BDNF, COPA, GADD45B, GPX7, ITGB5, LOX, MATN3, MCM7, MMP1, and SPP1, which were associated with different stages of tumor progression. BDNF is a widely studied biomarker in the nervous system and has been strongly associated with depression and Alzheimer's disease [25]. Coatomer subunit α (COPA), a protein-recoding editing target, was edited differently in HCC, as first evidenced by Song et al. [26]. The further study found that under-editing of COPA facilitates HCC progression via PI3K/AKT/mTOR signaling pathway [27]. GADD45B, belongs to the growth arrest and DNA damage-inducible 45 gene family, and is related to oncogenic stress, cell cycle arrest, and apoptosis [28]. However, the role of GADD45B in cancer remains unclear. The suppression of GADD45B represses cell invasion and migration of cholangiocarcinoma by regulating EMT [29]. Evidence has indicated that the up-regulated expression of GADD45B is correlated with an advanced stage and decreased OS in patients with colorectal cancer [30], and it may be an independent prognostic factor for patients with papillary thyroid carcinoma [31]. Our results demonstrated that the expression of GADD45B was down-regulated. This may be attributed to the inhibition of GADD45B autophagy and promotion of apoptosis [32]. GPX7 has been verified to be up-regulated in HCC tissues, and its high expression was associated with grade III–IV [33]. The abnormal expression of GPX7 is related to pathological conditions, for example, the deletion of GPX7 increases the differentiation of preadipocytes and the risk of cancer [34]. ITGB5 not only regulates the biological behavior of cancer through the tumor microenvironment but also plays an important role in stemness and chemotherapy resistance in cancer cells [35, 36]. LOX is highly expressed in patients with gastric cancer, indicating poor prognosis and possibly promoting the progression of cancer cells through ECM receptor I interaction and TGF-β, Wnt, JAK–STAT, and mTOR signaling pathways [37, 38]. MATN3, also known as EDM [22], is primarily responsible for homeostasis in vivo and carcinogenesis in various tumors [39]. As a prognostic factor in patients with HCC postoperatively, MCM7-positive are sensitive to sorafenib treatment [40]. MMP1 is composed of tumor cells and stromal cells, and it is well known that the over-expression of MMP1 promotes the migration and invasion of HCC cells [41]. In addition, the role of SPP1 in HCC tissues and cells has been widely explored, and the results suggest that it may be a potential target for prognosis and treatment [42, 43]. Seven genes of the prognostic model (COPA, GPX7, ITGB5, MATN3, MCM7, MMP1, and SPP1) have been validated to be related to tumor progression, whereas the remaining three genes, BNDF, GADD45B, and LOX, need to be further analyzed. Moreover, we investigated the mRNA expression of EMT-related prognostic signatures in HCC cells using qRT-PCR. The novel prognostic model of EMT-related gene signature could be a better biomarker if the mechanisms of these genes in patients with HCC are extensively investigated.

Previous studies have elucidated that immune microenvironment plays a momentous role in tumorigenesis [44–46]. However, we confirmed that a high-risk score is markedly associated with immune cell infiltration and function, specifically those of Treg, Th_cell, and macrophages, compared to a low-risk score. A high-risk score was also related to APC co-simulation, CCR, and type II IFN response. The increase in tumor-associated macrophages and Treg cells was correlated with poor prognosis in patients with HCC, as evidenced by Zhou et al. [47, 48]. Moreover, Shankaran et al. reported that type II IFN response combined with lymphocytes acts as an effective extrinsic tumor-suppressor system, prevents the development of spontaneous epithelial cancer, and selects tumor cells with reduced immunogenicity [49]. Immunotherapy in patients with HCC has greatly changed the mode of cancer treatment, especially immune checkpoint inhibitors [50]. We found that the expression of the majority of immune checkpoint genes was up-regulated in the high-risk group compared to the low-risk group and positively correlated with the risk score. Currently, programmed cell death protein 1 (PD-1) or its ligands PD-L1 and cytotoxic T-lymphocyte-associated protein 4 (CTLA-4) are approved for the treatment of cancer patients. Particularly, the activation of helper CD4 + T cells and initiation of the immune response mainly depend on CTLA-4 [51, 52]. In contrast, we investigated the relationship between immune infiltration subtype and risk score. Results have suggested that a high-risk score was related to subtypes C1 and C2, whereas a low-risk score was related to subtypes C3 and C4, indicating that subtypes C1 and C2 had worse prognosis than subtypes C3 and C4 in patients with HCC. Therefore, this study strongly verified the value of this signature for forecasting the HCC immune microenvironment.

This study has some limitations. First, to our knowledge, the clinical information contained in both the TCGA and ICGC databases is limited. Second, because this study was retrospective, the reliability of this model needs to be confirmed by prospective cohort studies in the future. Third, although the prognostic model is a reliable way to judge the prognosis of HCC, the potential mechanisms of these ten EMT-related genes and HCC need to be further verified by in vivo and in vitro experiments.

## Conclusion

We formed a novel signature of EMT-related prognostic genes that strongly predicted the prognosis of patients with HCC. The signature was proven to be absolutely linked to OS and clinicopathological characteristics in the training and validation cohorts. Furthermore, we found that the risk score structured by the model plays an essential role in the tumor microenvironment, functional enrichment analysis, and drug sensitivity. However, further prospective and multicenter cohorts to verify whether this signature contributes to the individualized treatment of patients with HCC are warranted.

## Supplementary Information

Below is the link to the electronic supplementary material.

Supplementary file1 (XLSX 17 KB)

Supplementary file2 (XLSX 9 KB)

Supplementary Figure 1 EMT-related prognostic genes expression between HCC and adjacent normal tissues in TCGA cohort. **A** BDNF. **B** COPA.** C** GADD45B. **D** GPX7. **E** ITGB5. **F** LOX. **G** MANT3. **H** MCM7. **I** MMP1. **J** SPP1. *P* < 0.05. Supplementary file3 (TIF 83438 KB)

Supplementary Figure 2 Survival analysis of EMT-related signature based on optimal cut-off expression value in TCGA cohort. **A** BDNF. **B** COPA. **C** GADD45B. **D** GPX7. **E** ITGB5. **F** LOX. **G** MANT3. **H** MCM7. **I** MMP1. **J** SPP1. *P* < 0.05.. Supplementary file4 (TIF 122000 KB)

Supplementary Figure 3 Analysis of PD-L1, CTLA4, CXCR2, TLR8 expression in high-risk and low-risk groups, and the correlation between risk score and its expression. **A**, **E** PD-L1. **B**, **F** CTLA4 **C**, **G** CXCR2. **D**, **H** TLR8.Supplementary file5 (TIF 57296 KB)

Supplementary Figure 4 Analysis of correlation between EMT-related gene expression and drug sensitivity. **A** GPX7. **B** MATN3. **C** GADD45B. **D** ITGB5. **E** MMP1. **F** BDNF. **G** LOX. Supplementary file6 (TIF 7081 KB)

## Data Availability

The datasets obtained and explored in this study are available in the TCGA and ICGC. (https://portal.gdc.cancer.gov/), (https://icgc.org/).

## Abbreviations

HCC: Hepatocellular carcinoma
ssGSEA: Single sample gene set enrichment analysis
EMT: Epithelial–mesenchymal transition
DEGs: Differential expressed genes
OS: Overall survival
TCGA: The Cancer Genome Atlas
ICGC: The International Cancer Genome Consortium
LASSO: Least absolute shrinkage and selection operator
ROC: Receiver-operating characteristic
PCA: Principal component analyses
t-SNE: T-distributed random neighbor embedding analysis
TME: Tumor microenvironment
RNAss: RNA stemness score
DNAss: DNA stemness score
GO: Gene ontology
KEGG: Kyoto encyclopedia of genes and genomes

## References

[1] Sung H, Ferlay J, Siegel RL, Laversanne M, Soerjomataram I, Jemal A, Bray F (2021). Global Cancer Statistics 2020: GLOBOCAN estimates of incidence and mortality worldwide for 36 cancers in 185 countries. CA Cancer J Clin.

[2] Yang JD, Hainaut P, Gores GJ, Amadou A, Plymoth A, Roberts LR (2019). A global view of hepatocellular carcinoma: trends, risk, prevention and management. Nat Rev Gastroenterol Hepatol.

[3] Jiang Y, Xie J, Han Z, Liu W, Xi S, Huang L, Huang W, Lin T, Zhao L, Hu Y (2018). Immunomarker support vector machine classifier for prediction of gastric cancer survival and adjuvant chemotherapeutic benefit. Clinical cancer research : an official journal of the American Association for Cancer Research.

[4] DiStefano JK, Davis B (2019). Diagnostic and prognostic potential of AKR1B10 in human hepatocellular carcinoma. Cancers.

[5] Greenburg G, Hay ED (1982). Epithelia suspended in collagen gels can lose polarity and express characteristics of migrating mesenchymal cells. J Cell Biol.

[6] Baulida J (2017). Epithelial-to-mesenchymal transition transcription factors in cancer-associated fibroblasts. Mol Oncol.

[7] Shibue T, Weinberg RA (2017). EMT, CSCs, and drug resistance: the mechanistic link and clinical implications. Nat Rev Clin Oncol.

[8] Qiu GZ, Jin MZ, Dai JX, Sun W, Feng JH, Jin WL (2017). Reprogramming of the tumor in the hypoxic niche: the emerging concept and associated therapeutic strategies. Trends Pharmacol Sci.

[9] Calvisi DF (2015). When good transforming growth factor-β turns bad in hepatocellular carcinoma: Axl takes the stage. Hepatology (Baltimore, MD).

[10] Xiao T, Zhang Q, Zong S, Zhong WL, Qin Y, Bi Z, Chen S, Liu HJ, Wei JJ, Zhou BJ (2018). Protease-activated receptor-1 (PAR1) promotes epithelial-endothelial transition through Twist1 in hepatocellular carcinoma. J Exp Clin Cancer Res CR.

[11] Zhao L, Jiang L, He L, Wei Q, Bi J, Wang Y, Yu L, He M, Zhao L, Wei M (2019). Identification of a novel cell cycle-related gene signature predicting survival in patients with gastric cancer. J Cell Physiol.

[12] Lin Z, Xu Q, Miao D, Yu F (2021). An inflammatory response-related gene signature can impact the immune status and predict the prognosis of hepatocellular carcinoma. Front Oncol.

[13] Friedman J, Hastie T, Tibshirani R (2010). Regularization paths for generalized linear models via coordinate descent. J Stat Softw.

[14] Tibshirani R (1997). The lasso method for variable selection in the Cox model. Stat Med.

[15] Liu C, Wang X, Genchev GZ, Lu H (2017). Multi-omics facilitated variable selection in Cox regression model for cancer prognosis prediction. Methods (San Diego, CA).

[16] Desgrosellier JS, Cheresh DA (2010). Integrins in cancer: biological implications and therapeutic opportunities. Nat Rev Cancer.

[17] Dai Y, Qiang W, Lin K, Gui Y, Lan X, Wang D (2021). An immune-related gene signature for predicting survival and immunotherapy efficacy in hepatocellular carcinoma. Cancer Immunol Immunother CII.

[18] Deng T, Hu B, Jin C, Tong Y, Zhao J, Shi Z, Zhang T, Deng L, Sun Z, Chen G (2021). A novel ferroptosis phenotype-related clinical-molecular prognostic signature for hepatocellular carcinoma. J Cell Mol Med.

[19] Hodorogea A, Calinescu A, Antohe M, Balaban M, Nedelcu RI, Turcu G, Ion DA, Badarau IA, Popescu CM, Popescu R (2019). Epithelial-mesenchymal transition in skin cancers: a review. Anal Cell Pathol (Amst).

[20] Tao L, Shu-Ling W, Jing-Bo H, Ying Z, Rong H, Xiang-Qun L, Wen-Jie C, Lin-Fu Z (2020). MiR-451a attenuates doxorubicin resistance in lung cancer via suppressing epithelial–mesenchymal transition (EMT) through targeting c-Myc. Biomed Pharmacother.

[21] Osorio LA, Farfán NM, Castellón EA, Contreras HR (2016). SNAIL transcription factor increases the motility and invasive capacity of prostate cancer cells. Mol Med Rep.

[22] Zhang M, Du H, Wang L, Yue Y, Zhang P, Huang Z, Lv W, Ma J, Shao Q, Ma M (2020). Thymoquinone suppresses invasion and metastasis in bladder cancer cells by reversing EMT through the Wnt/β-catenin signaling pathway. Chem Biol Interact.

[23] Yang L, Qiu J, Xiao Y, Hu X, Liu Q, Chen L, Huang W, Li X, Li L, Zhang J (2018). AP-2β inhibits hepatocellular carcinoma invasion and metastasis through Slug and Snail to suppress epithelial–mesenchymal transition. Theranostics.

[24] Sanghera C, Teh JJ, Pinato DJ (2019). The systemic inflammatory response as a source of biomarkers and therapeutic targets in hepatocellular carcinoma. Liver Int.

[25] Murawska-Ciałowicz E, Wiatr M, Ciałowicz M, Gomes de Assis G, Borowicz W, Rocha-Rodrigues S, Paprocka-Borowicz M, Marques A (2021). BDNF impact on biological markers of depression-role of physical exercise and training. Int J Environ Res Public Health.

[26] Chan TH, Lin CH, Qi L, Fei J, Li Y, Yong KJ, Liu M, Song Y, Chow RK, Ng VH (2014). A disrupted RNA editing balance mediated by ADARs (adenosine deaminases that act on RNA) in human hepatocellular carcinoma. Gut.

[27] Song Y, An O, Ren X, Chan THM, Tay DJT, Tang SJ, Han J, Hong H, Ng VHE, Ke X (2021). RNA editing mediates the functional switch of COPA in a novel mechanism of hepatocarcinogenesis. J Hepatol.

[28] Liebermann DA, Tront JS, Sha X, Mukherjee K, Mohamed-Hadley A, Hoffman B (2011). Gadd45 stress sensors in malignancy and leukemia. Crit Rev Oncog.

[29] Myint KZ, Kongpracha P, Rattanasinganchan P, Leelawat K, Moolthiya P, Chaiyabutr K, Tohtong R (2018). Gadd45β silencing impaired viability and metastatic phenotypes in cholangiocarcinoma cells by modulating the EMT pathway. Oncol Lett.

[30] Zhao Z, Gao Y, Guan X, Liu Z, Jiang Z, Liu X, Lin H, Yang M, Li C, Yang R (2018). GADD45B as a prognostic and predictive biomarker in stage II colorectal cancer. Genes.

[31] Barros-Filho MC, de Mello JBH, Marchi FA, Pinto CAL, da Silva IC, Damasceno PKF, Soares MBP, Kowalski LP, Rogatto SR (2020). GADD45B transcript is a prognostic marker in papillary thyroid carcinoma patients treated with total thyroidectomy and radioiodine therapy. Front Endocrinol.

[32] Chen Z, Wan X, Hou Q, Shi S, Wang L, Chen P, Zhu X, Zeng C, Qin W, Zhou W (2016). GADD45B mediates podocyte injury in zebrafish by activating the ROS-GADD45B-p38 pathway. Cell Death Dis.

[33] Guerriero E, Capone F, Accardo M, Sorice A, Costantini M, Colonna G, Castello G, Costantini S (2015). GPX4 and GPX7 over-expression in human hepatocellular carcinoma tissues. Eur J Histochem EJH.

[34] Buday K, Conrad M (2021). Emerging roles for non-selenium containing ER-resident glutathione peroxidases in cell signaling and disease. Biol Chem.

[35] Hamidouche Z, Fromigué O, Ringe J, Häupl T, Vaudin P, Pagès JC, Srouji S, Livne E, Marie PJ (2009). Priming integrin alpha5 promotes human mesenchymal stromal cell osteoblast differentiation and osteogenesis. Proc Natl Acad Sci USA.

[36] Xiao Y, Li Y, Tao H, Humphries B, Li A, Jiang Y, Yang C, Luo R, Wang Z (2018). Integrin α5 down-regulation by miR-205 suppresses triple negative breast cancer stemness and metastasis by inhibiting the Src/Vav2/Rac1 pathway. Cancer Lett.

[37] Farhat A, Ferns GA, Ashrafi K, Arjmand MH (2021). Lysyl oxidase mechanisms to mediate gastrointestinal cancer progression. Gastrointest Tumors.

[38] Zhu J, Luo C, Zhao J, Zhu X, Lin K, Bu F, Yu Z, Zou F, Zhu Z (2021). Expression of LOX suggests poor prognosis in gastric cancer. Front Med.

[39] Yiqi Z, Ziyun L, Qin F, Xingli W, Liyu Y (2020). Identification of 9-gene epithelial-mesenchymal transition related signature of osteosarcoma by integrating multi cohorts. Technol Cancer Res Treat.

[40] Zhou YM, Zhang XF, Cao L, Li B, Sui CJ, Li YM, Yin ZF (2012). MCM7 expression predicts post-operative prognosis for hepatocellular carcinoma. Liver Int.

[41] Yu CL, Yu YL, Yang SF, Hsu CE, Lin CL, Hsieh YH, Chiou HL (2021). Praeruptorin A reduces metastasis of human hepatocellular carcinoma cells by targeting ERK/MMP1 signaling pathway. Environ Toxicol.

[42] Castello LM, Raineri D, Salmi L, Clemente N, Vaschetto R, Quaglia M, Garzaro M, Gentilli S, Navalesi P, Cantaluppi V (2017). Osteopontin at the crossroads of inflammation and tumor progression. Mediators Inflamm.

[43] Nardo AD, Grün NG, Zeyda M, Dumanic M, Oberhuber G, Rivelles E, Helbich TH, Markgraf DF, Roden M, Claudel T (2020). Impact of osteopontin on the development of non-alcoholic liver disease and related hepatocellular carcinoma. Liver Int.

[44] Pereira MB, Barros LRC, Bracco PA, Vigo A, Boroni M, Bonamino MH, Lenz G (2018). Transcriptional characterization of immunological infiltrates and their relation with glioblastoma patients overall survival. Oncoimmunology.

[45] Lei X, Lei Y, Li JK, Du WX, Li RG, Yang J, Li J, Li F, Tan HB (2020). Immune cells within the tumor microenvironment: Biological functions and roles in cancer immunotherapy. Cancer Lett.

[46] Piñeiro Fernández J, Luddy KA, Harmon C, O’Farrelly C (2019). Hepatic tumor microenvironments and effects on NK cell phenotype and function. Int J Mol Sci.

[47] Zhou SL, Zhou ZJ, Hu ZQ, Huang XW, Wang Z, Chen EB, Fan J, Cao Y, Dai Z, Zhou J (2016). Tumor-associated neutrophils recruit macrophages and T-regulatory cells to promote progression of hepatocellular carcinoma and resistance to sorafenib. Gastroenterology.

[48] Fu J, Xu D, Liu Z, Shi M, Zhao P, Fu B, Zhang Z, Yang H, Zhang H, Zhou C (2007). Increased regulatory T cells correlate with CD8 T-cell impairment and poor survival in hepatocellular carcinoma patients. Gastroenterology.

[49] Shankaran V, Ikeda H, Bruce AT, White JM, Swanson PE, Old LJ, Schreiber RD (2001). IFNgamma and lymphocytes prevent primary tumour development and shape tumour immunogenicity. Nature.

[50] Zhou G, Sprengers D, Boor PPC, Doukas M, Schutz H, Mancham S, Pedroza-Gonzalez A, Polak WG, de Jonge J, Gaspersz M (2017). Antibodies against immune checkpoint molecules restore functions of tumor-infiltrating T cells in hepatocellular carcinomas. Gastroenterology.

[51] Han Y, Chen Z, Yang Y, Jiang Z, Gu Y, Liu Y, Lin C, Pan Z, Yu Y, Jiang M (2014). Human CD14+ CTLA-4+ regulatory dendritic cells suppress T-cell response by cytotoxic T-lymphocyte antigen-4-dependent IL-10 and indoleamine-2,3-dioxygenase production in hepatocellular carcinoma. Hepatology (Baltimore, MD).

[52] Giannini EG, Aglitti A, Borzio M, Gambato M, Guarino M, Iavarone M, Lai Q, Levi Sandri GB, Melandro F, Morisco F (2019). Overview of immune checkpoint inhibitors therapy for hepatocellular carcinoma, and the ITALICA cohort derived estimate of amenability rate to immune checkpoint inhibitors in clinical practice. Cancers.

