# Supplementary figures and images for: A novel epithelial–mesenchymal transition gene signature for the immune status and prognosis of hepatocellular carcinoma
Source: Hepatol Int. 2022 Jun 14;16(4):906–17. doi: 10.1007/s12072-022-10354-3 (PMC9349121; doi:10.1007/s12072-022-10354-3)

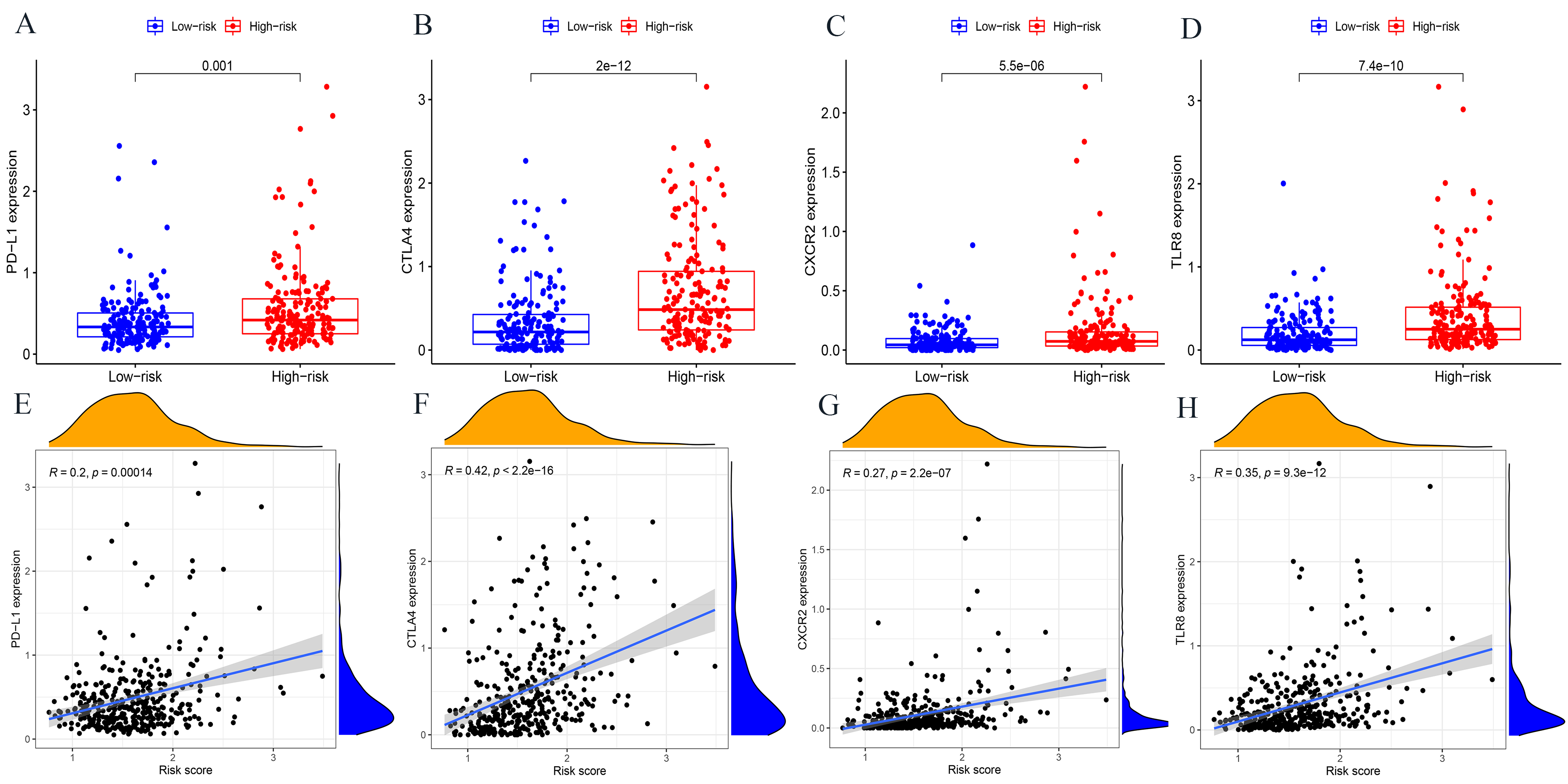

Supplement: Supplementary file 5 — Analysis of PD-L1, CTLA4, CXCR2, TLR8 expression in high-risk and low-risk groups, and the correlation between risk score and its expression. A, E PD-L1. B, F CTLA4 C, G CXCR2. D, H TLR8.Supplementary file5 (TIF 57296 KB) [file 12072_2022_10354_MOESM5_ESM.tif]

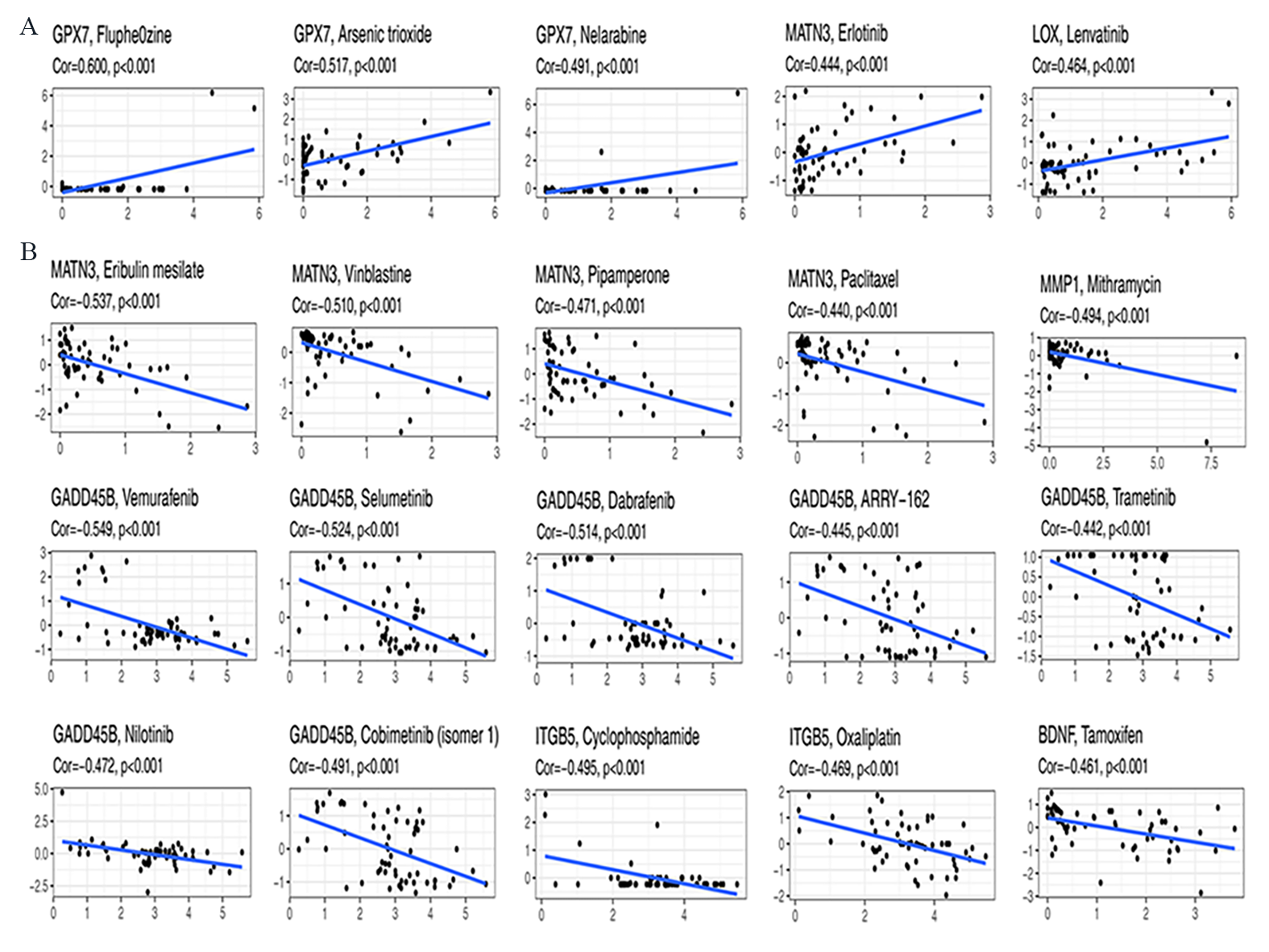

Supplement: Supplementary file 6 — Analysis of correlation between EMT-related gene expression and drug sensitivity. A GPX7. B MATN3. C GADD45B. D ITGB5. E MMP1. F BDNF. G LOX. Supplementary file6 (TIF 7081 KB) [file 12072_2022_10354_MOESM6_ESM.tif]
